# Night Heart Rate Variability and Particulate Exposures among Boilermaker Construction Workers

**DOI:** 10.1289/ehp.10019

**Published:** 2007-04-05

**Authors:** Jennifer M. Cavallari, Ellen A. Eisen, Jiu-Chiuan Chen, Shona C. Fang, Christine B. Dobson, Joel Schwartz, David C. Christiani

**Affiliations:** 1 Department of Environmental Health, Harvard School of Public Health, Boston, Massachusetts, USA; 2 Department of Epidemiology, UNC School of Public Health, Chapel Hill, North Carolina, USA; 3 Harvard Medical School, Boston, Massachusetts, USA; 4 Massachusetts General Hospital, Boston, Massachusetts, USA

**Keywords:** environmental cardiology, heart rate variability, occupational, particulate exposures, welders

## Abstract

**Background:**

Although studies have documented the association between heart rate variability (HRV) and ambient particulate exposures, the association between HRV, especially at night, and metal-rich, occupational particulate exposures remains unclear.

**Objective:**

Our goal in this study was to investigate the association between long-duration HRV, including nighttime HRV, and occupational PM_2.5_ exposures.

**Methods:**

We used 24-hr ambulatory electrocardiograms (ECGs) to monitor 36 male boilermaker welders (mean age of 41 years) over a workday and nonworkday. ECGs were analyzed for HRV in the time domain; rMSSD (square root of the mean squared differences of successive intervals), SDNN (SD of normal-to-normal intervals over entire recording), and SDNN_i_ (SDNN for all 5-min segments) were summarized over 24-hr, day (0730–2130 hours), and night (0000–0700 hours) periods. PM_2.5_ (particulate matter with an aerodynamic diameter ≤ 2.5 μm) exposures were monitored over the workday, and 8-hr time-weighted average concentrations were calculated. We used linear regression to assess the associations between HRV and workday particulate exposures. Matched measurements from a nonworkday were used to control for individual cardiac risk factors.

**Results:**

Mean (± SD) PM_2.5_ exposure was 0.73 ± 0.50 mg/m^3^ and ranged from 0.04 to 2.70 mg/m^3^. We observed a consistent inverse exposure–response relationship, with a decrease in all HRV measures with increased PM_2.5_ exposure. However, the decrease was most pronounced at night, where a 1-mg/m^3^ increase in PM_2.5_ was associated with a change of −8.32 [95% confidence interval (CI), −16.29 to −0.35] msec nighttime rMSSD, −14.77 (95% CI, −31.52 to 1.97) msec nighttime SDNN, and −8.37 (95% CI, −17.93 to 1.20) msec nighttime SDNN_i_, after adjusting for nonworking nighttime HRV, age, and smoking.

**Conclusion:**

Metal-rich particulate exposures were associated with decreased long-duration HRV, especially at night. Further research is needed to elucidate which particulate metal constituent is responsible for decreased HRV.

Although it has been established that welders experience acute and chronic pulmonary health effects, welders may also experience cardiovascular effects. Several mortality studies have reported increased risk of death due to ischemic heart disease among welders (Moulin et al. 1993; Newhouse et al. 1985; Sjogren et al. 2002). In addition, an increased risk of myocardial infarction and higher prevalence of angina pectoris has also been reported among welders (Hilt et al. 1999).

Welders’ primary occupational exposure is welding fume, a metal-rich mixture of fine and ultrafine particulate matter (Glinsmann and Rosenthal 1985). Welding is part of the normal work duties for a number of workers, including boilermaker construction workers (or “boilermakers”) who build, maintain, and repair industrial boilers, vessels, and tanks that are primarily located at power plants. Boilermakers are occupationally exposed to welding fume and the residual ash from the combustion products specific to the power plant’s fuel source. Although previous studies confirm high levels of metal-rich particulate exposures among boilermakers (Kim et al. 2004; Liu et al. 2005), it is unclear what role these exposures play in the development of cardiovascular outcomes among welders.

However, studies of ambient particulate air pollution provide plausible evidence of numerous pathophysiologic pathways of particulate-induced cardiovascular effects, including one pathway that involves altered cardiac autonomic function (Brook et al. 2003, 2004; Pope and Dockery 2006; Schulz et al. 2005; Utell et al. 2002). Studies examining the association between particulates and changes in cardiac autonomic function have used heart rate variability (HRV) as a measure of cardiovascular autonomic control. Decreased HRV has been linked to adverse cardiovascular outcomes, including increased risk of death among chronic heart failure patients (La Rovere et al. 2003; Ponikowski et al. 1997) and onset of hypertension (Schroeder et al. 2003; Singh et al. 1998), and is also a predictor of the rapid progression of atherosclerosis (Huikuri et al. 1999). Several review articles have described consistent associations of decreased HRV with particulate exposures (Brook et al. 2003, 2004; Peters 2005; Pope and Dockery 2006; Schulz et al. 2005). *In vivo* studies support the epidemiologic studies (Chang et al. 2005; Chen and Hwang 2005; Corey et al. 2006; Lippmann et al. 2005; Rodriguez Ferreira Rivero et al. 2005) and further suggest that the transition metal components of particulate matter may be responsible for particulate-induced cardiovascular changes (Campen et al. 2001, 2002; Wellenius et al. 2002); this lends additional evidence for the cardiotoxicity of welding fume.

Epidemiologic studies have used various methods to study the HRV-particulate association, in part, because of uncertainty in the mechanism(s) or time course of HRV responses to particulate exposures. Studies have used both short-term HRV (often 5 min) and long-term HRV (usually in hours, most commonly 24 hr) with particulate exposures that differ in both duration and lag time. The majority of epidemiologic studies have examined changes in short-duration HRV with particulate exposures averaged over 2–24 hr, with lags ranging from 0 to 3 days, as reviewed by Schulz et al. (2005) and Pope and Dockery (2006). In fact, previous investigations of boilermakers found that 4-hr mean occupational exposures to PM_2.5_ (particulate matter with an aerodynamic diameter ≤ 2.5 μm) are associated with changes in short-duration HRV, 5 min SDNN (Magari et al. 2001). However, to date, few studies have examined the association between particulate exposures and long-duration HRV (Brauer et al. 2001; Magari et al. 2002; Pope et al. 1999, 2004; Vallejo et al. 2006) and only one study reported long-duration, nighttime HRV (Pope et al. 1999).

Using a cohort of boilermakers with continuous 24-hr ambulatory electrocardiography (ECG) monitoring over both a workday and a nonworkday, this crossover panel study sought to expand upon the investigation of HRV responses to metal-rich particulate by examining the association between particulate exposures and HRV measured over the entire 24-hr day, as well as divided between the 14-hr day and 7-hr night periods. The crossover design accounted for the inherent heterogeneity between individuals in personal characteristics, including cardiac risk factors, as nonworkday monitoring provided a baseline measure of HRV on a day when particulate exposures are low compared with workday levels. We chose long-duration HRV measures, in part, because they have been shown to be less influenced by daily activities including physical activity (Task Force of the European Society of Cardiology and the North American Society of Pacing and Electrophysiology 1996) and more stable over time with low within-person day-to-day variation (Kleiger et al. 1991). *A priori*, we hypothesized that HRV at night would better capture the time course of the biological response, consistent with previous evidence of a lag (Pope et al. 1999). Night, a period dominated by sleep, is often free from physical activity, which is a strong predictor of HRV and a potential confounder. Studies suggest that sleep may be a condition in which HRV best identifies autonomic derangements because sleep is a condition in which cardiac autonomic activity can be studied in the absence of factors including physical activity and higher cortical factors (Vanoli et al. 1995). There is also clinical significance in understanding changes in HRV at night and during sleep. Despite the fact that sleep is a time when an individual is protected from known precipitating factors for cardiac events, 12–15% of all cardiac events and almost 36,000 deaths annually occur during sleep, resulting in a substantial health problem and raising the question of mechanism (Muller et al. 1997). We hypothesized that workday particulate exposures would be associated with declines in 24-hr, day and night HRV and that night would best capture this association among the boilermaker cohort.

## Methods

### Subject recruitment

The Institutional Review Board at the Harvard School of Public Health approved the study protocol, and informed consent was obtained from each adult prior to participation. From 1999 to 2006, we recruited 64 male boilermakers from a local union into a panel study to assess cardiac responses to particulates. Workers were recruited while training at an apprentice welding school and while working on the repair and maintenance of an oil-fired boiler. Recruitment at the welding school occurred in the winter and summer months, when the majority of workers were not actively working. We solicited a random subset of 36 participants for extensive ECG monitoring over two 24-hr periods on both a workday and a non-workday. Workers participated in the study on multiple occasions over the 7-year sampling period; 26 (72%) were monitored on one occasion and 10 (28%) on multiple occasions. Workday and nonworkday monitoring occurred at each occasion.

### Data collection

Workday monitoring occurred at a union welding school and at the overhaul of an oil-fired boiler within a power plant. The welding school consisted of a large, temperature-controlled room outfitted with 10 workstations with local exhaust ventilation where the boilermakers received instruction and practiced welding, cutting, and grinding techniques. The boiler overhaul entailed removing and replacing the boiler’s interior wall panels and water-circulating tubing. At both locations, boilermakers primarily performed shielded metal arc welding (stick) and gas metal arc welding, most commonly using base metals of mild steel (manganese alloys) and stainless steel (manganese, chromium, and nickel alloys) with electrodes composed mainly of iron with variable amounts of manganese (1–5%). Plasma arc or acetylene torch cutting and grinding also occurred at both work sites.

Participants were also monitored over a 24-hr period on a nonworkday when they were not welding, grinding, or cutting. Although nonworkday monitoring occurred within 6 months of the workday monitoring, 80% of the observations occurred within the same week as the workday monitoring.

We used a self-administered questionnaire to collect information on medical history and medication use for each participation year. Participants were classified as cardiac compromised if they reported any of the following: heart trouble, congestive heart failure, angina, arrhythmia, myocardial infarction, heart/chest operation, or otherwise nonclassified heart problems. Hypertension was classified by reporting a doctor diagnosis or the use of hypertensive medications including ACE (angiotensin-converting enzyme) inhibitors or beta-blockers. We also collected demographic and lifestyle information, including smoking and occupational history.

### Particulate exposure assessment

We continuously monitored personal, cross-shift PM_2.5_ exposures using the light-scattering technology of a DustTrak Aerosol Monitor (TSI Inc., St. Paul, MN) fitted with a PM_2.5_ inlet impactor. The monitor was placed in a padded pouch, with the inlet tubing secured to the participant’s shoulder in the breathing zone area. The DustTrak received yearly factory calibration and daily zero balance and flow checks. The monitor took PM_2.5_ concentration readings every 10 sec and recorded 1-min averages. Participants were asked to keep a work log to record information on shift length and respirator use. We calculated 8-hr time-weighted average (TWA_8-hr_) PM_2.5_ exposures from the 1-min PM_2.5_ concentrations over the reported shift length. A previous study of welding fume exposures (Kim et al. 2004) validated the use of the DustTrak to capture welding fume exposures.

### Continuous Holter monitoring and tape processing

Participants were fitted with a standard 5-lead ECG Holter monitor. To facilitate good lead contacts, the participant’s skin was shaved (if necessary), cleansed with an alcohol wipe, and slightly abraded. At the workplace, study staff periodically checked leads. Each 24-hr tape was sent to Raytel Cardiac Services (Haddonfield, NJ) for processing and analysis using a StrataScan 563 (DelMar Avionics, Irvine, CA). Only beats with an RR interval between 0.6 and 1.5 sec and an RR ratio of 0.8–1.2 were included in the analysis. Trained technicians, blinded to work and nonwork periods and exposures, used standard criteria to accept or reject all normal or abnormal findings. Tapes were analyzed in the time domain, and indexes including the square root of the mean of the sum of the squared differences between adjacent NN intervals (rMSSD), the SD of all NN intervals over the entire period (SDNN), and the mean of the SDNN intervals for all 5-min segments (SDNN_i_) were calculated over the entire 24-hr recording, the 14-hr day period (0730–2130 hours), and the 7-hr night period (0000 to 0700 hours).

### Data analysis

To account for correlated outcomes among subjects who participated on multiple occasions, we used mixed-effects regression models with random intercepts and unstructured covariance to investigate the association between each of the HRV outcomes and either dichotomous or continuous PM_2.5_ exposure. For dichotomous exposure, the outcomes of interest were work and non-work HRV measures, and the predictors included a dichotomous exposure variable (work/nonwork) and a random intercept for individuals. The *p*-value of the dichotomous exposure regression coefficient was used to assess statistically significant differences between work and nonwork HRV measures. Next, continuous workday PM_2.5_ exposures were investigated by modeling each workday HRV outcome while adjusting for nonwork-day HRV measured at the same time scale and during the same period as the workday outcome. Because baseline, nonwork HRV was included in each model, personal factors (including cardiac risk factors) that do not change between work and nonwork measurements were unlikely to introduce bias due to confounding. However, because age and smoking are strong predictors of HRV, they were included in each model as a continuous and dichotomous covariate, respectively. Each outcome measure (rMSSD, SDNN, and SDNN_i_) summarized over the three time periods (24-hr, 14-hr day, and 7-hr night) gave a total of nine exposure–response models.

We further investigated model results by restricting the cohort. Because of small sample size and unbalanced design, a formal investigation of effect modification was not feasible. However, we compared the effect estimates from the full cohort to a restricted sample of participants who did not report cardiac compromise or hypertension at the workday measurement to evaluate whether the exposure–response relationship varied by health conditions.

We assessed distributional assumptions by examining residuals plotted against predictors. We used Cook’s distance to identify potentially influential points using a criteria of Cook’s distance > 0.5. Statistical significance was assessed at the α = 0.05 level in two-sided tests. All analyses were performed using PROC MIXED in SAS version 9.1 (SAS Institute Inc., Cary, NC).

## Results

The study population consisted of 36 males, with a mean age of 41 years; 29 (81%) of the participants were white (Table 1). Of the 36 participants, 10 contributed more than one work/nonwork pair of observations. Eight were monitored twice, 1 was monitored on three occasions, and 1 was monitored on four occasions. There were 12 smokers at study enrollment, and 2 quit smoking by subsequent participation. Five participants reported cardiac conditions, including two myocardial infarctions, one stent, one murmur, and one arrhythmia. Three of these individuals participated only once, and two participated twice, reporting no cardiac condition on first participation. Six participants reported either doctor diagnosis of hypertension or were using medication for hypertension. Of these six individuals, three participated once, one twice (hypertension reported both occasions), one three times (hypertension reported two occasions), and one four times (hypertension reported at one occasion). Two participants reported the use of statins, and three used ACE inhibitors; no one reported using beta-blockers, calcium channel blockers, or diuretics. TWA_8-hr_ PM_2.5_ exposures ranged from 0.04 to 2.70 mg/m^3^ with a mean (± SD) of 0.73 ± 0.50 mg/m^3^ (Table 1). Workers reported respirator use on 13 (26%) monitoring occasions. Forty-five (92%) of the 49 monitored workdays occurred at the apprentice school.

Ambulatory ECG data were collected for a total of 98 person-days and are summarized in Table 2. The mean levels of the 24-hr HRV measures were unremarkable and were comparable to previously reported levels of rMSSD (35 ± 11 msec), SDNN (146 ± 30 msec), and SDNN_i_ (58 ± 18 msec) for men 40–49 years of age (Jensen-Urstad et al. 1997). We found a wide range of HRV within each period over both workdays and nonworkdays because of between-person heterogeneity. However, the mean work rMSSD and SDNN_i_ measures were consistently lower than the mean non-work HRV measures, and statistically significant differences were found between work and nonwork across all time periods for rMSSD and SDNN_i_ measures, with the exception of night rMSSD (Table 2). SDNN measures across the three time periods did not follow this pattern, and in fact, there was no statistical difference between work and nonwork SDNN across all time periods.

The mixed model regression analyses revealed that elevated PM_2.5_ levels were consistently associated with declines in rMSSD, SDNN, and SDNN_i_ over the 24-hr, day, and night periods after adjusting for HRV during matched time-periods of nonworkdays, age, and smoking status (Table 3). When the 24-hr period was broken into day and night, the most pronounced decline in HRV was during the night, where statistically significant associations were observed for night rMSSD.

We further explored the data by performing a series of restricted analyses for each model after adjusting for nonwork nighttime HRV, age, and smoking status (Table 4). In the cohort restricted to participants reporting no cardiac compromise (model II) or among those who did not report or were not on medication for hypertension (model III), the declines in HRV at night subsequent to workday particulate exposures were comparable to results from the unrestricted cohort (model I). Examination of residual plots and Cook’s distance confirmed model assumptions and showed no evidence of influential points for all models.

## Discussion

Among a cohort of boilermakers, we observed statistically significant lower 24-hr, day and night rMSSD and SDNN_i_ on workdays compared with nonworkdays. Furthermore, we found an inverse dose–response relationship between workday PM_2.5_ exposure and 24-hr, day and night HRV. When the 24-hr period was separated into the day and night periods, the most pronounced declines in HRV were seen at night. This association was statistically significant for night rMSSD; every 1-mg/m^3^ increase in workday PM_2.5_ TWA_8-hr_ was associated with a change of −8.32 [95% confidence interval (CI), −16.29 to −0.35] msec nighttime rMSSD after adjusting for nonwork nighttime rMSSD, age, and smoking status. Few studies have documented the association between particulate exposures and long-duration HRV (Brauer et al. 2001; Magari et al. 2002; Pope et al. 1999, 2004; Vallejo et al. 2006), and to date, the majority have investigated the effect of ambient particulate air pollution exposures. The present study is the first to demonstrate declines in HRV over the entire day and night subsequent to occupational particulate exposures.

Because of differences in exposure characterization and HRV measures, direct comparison of our study results with previous studies is not feasible. However, the direction of associations in the present study is consistent with the inverse exposure–response relationship found in studies of particulate air pollution. Among a panel of seven compromised individuals, elevated PM_10_ (particulate matter with an aerodynamic diameter ≤ 10 μm) levels were associated with decreased 24-hr SDNN and increased 24-hr rMSSD (Pope et al. 1999). A subsequent study among 88 elderly Utah residents confirmed the inverse association between 24-hr SDNN and ambient particulate exposure, but also reported a decline in 24-hr rMSSD with PM_2.5_ exposures (Pope et al. 2004). A similar association was observed with 24-hr SDNN and rMSSD in a population of elderly individuals with chronic obstructive pulmonary disease (COPD) (Brauer et al. 2001). In addition to elderly and compromised individuals, an inverse association (between 2-hr HRV and 2-hr PM_2.5_ exposure) was observed among a young cohort of nonsmoking, healthy adults (Vallejo et al. 2006). These results are consistent with the declines in 24-hr SDNN_i_, SDNN, and rMSSD that we observed in the boilermaker cohort following PM_2.5_ exposure.

Likewise, the pattern of day and night long-duration HRV changes among this cohort is confirmed by air pollution studies. Pope et al. (1999) further analyzed the 24-hr HRV recordings by breaking them into 6-hr segments. The largest declines in HRV with PM_10_ exposures were during the late night (0000–0600) and morning (0600–1200) hours. In the present study, we also found heterogeneity among the effect estimates for 14-hr day and 7-hr night periods, with the largest declines seen at night. This larger and more statistically significant decline observed for nighttime HRV may be due to any of at least three possible explanations. First, the association between workday particulate exposures and nighttime HRV may capture the biologically relevant time course of the response. In our previous investigation among boilermakers, we observed no association between 8–10-hr workday SDNN_i_ and occupational PM_2.5_ exposures (Magari et al. 2002). The lack of association may be explained by not accounting for the appropriate lag between exposure and response. Lags of 24 hr have been suggested in studies of long-duration HRV and air pollution exposures where, compared with same-day PM_10_, a larger decrease in 24-hr SDNN and SDANN are found with previous-day PM_10_ (Pope et al. 1999). Although the lag between exposure (during the workday) and outcome (during the nighttime) was less than 24 hr in the present study, it is still biologically plausible that responses occur within a range of time from exposures. This is supported by an *in vivo* study among ApoE^−/−^ mice exposed to concentrated air pollutants (CAPs); following daytime (0900–1500 hours) exposure to CAPs, the largest decreases in rMSSD and SDNN occurred in the late night (0130–0430 hours) as compared to the afternoon (1600–1800 hours) or during exposure (Lippmann et al. 2005).

Second, HRV at night covers the sleep period, and the observed declines in nighttime HRV following particulate exposures may be related to sleep-state–dependent fluctuations in autonomic nervous system activity. Sleep is an important modulator of cardiovascular function (Wolk et al. 2005), with changes in HRV specific to the stages of sleep (Vaughn et al. 1995). In the present study, we were unable to account for sleep parameters; thus, the observed declines in HRV may result from exposure-related changes in sleep stage or quality. Disruption of sleep parameters is concerning; Wolk et al. (2005) suggested that sleep dynamics may play a role in the diurnal periodicity in the occurrence of cardiac arrhythmias and sudden death. Additional research is needed to determine how the nighttime HRV declines within this cohort are related to sleep-related fluctuations in HRV.

Third, night is a period free from potential confounders—predictors of HRV that may be correlated with daytime exposure, such as physical activity. During the day, when exposure and outcome are being measured over the same time period, the potential for confounding bias is greater than at night when there is a lag between workday exposures and nighttime HRV. Although the reasons remain unclear, the robust and statistically significant association between nighttime HRV and particulate exposure make night an optimal time to investigate the cardiovascular effects of workday particulate exposures.

The exposure characteristics of this cohort offered both a strength and limitation for this study. Occupational exposures offer a unique opportunity to investigate particulate-induced cardiovascular effects because particulate levels tend to be higher than ambient air pollution and occur over a discrete time period. However, these studies are complicated by the varying and often unmeasured composition of the exposure, which may be a source for the heterogeneity of results among similar occupational studies studying HRV and PM_2.5_ exposure. As in the present study, Magari et al. (2001) observed an inverse association between short-term HRV with PM_2.5_ exposure among a similar cohort of boilermakers exposed to metal-rich welding fume. In contrast, Riediker et al. (2004) reported that highway patrol troopers exposed to traffic-related particles demonstrated increased next-morning short-duration HRV following a night shift, and Eninger and Rosenthal (2004) found no association between end-of-shift, short-duration HRV parameters and particulate exposures among vehicle maintenance workers exposed to diesel. Toxicologic and epidemiologic evidence suggest that the metal component of particulate matter may play a role in particulate-induced cardiovascular effects (Schwarze et al. 2006) and that the metal-rich composition of welding fume may be responsible for the decreased HRV observed within this cohort. Future research should elucidate whether declines in nighttime HRV are related to particulate composition and level.

The unique metal-rich particulate exposures may limit the generalizability of the study both to occupational and community cohorts. The exposure concentrations within this cohort were higher than community-based ambient particulate levels and of different composition and temporal pattern. Mean workday concentrations were 1.14 mg/m^3^, as compared with reported 24-hr, ambient PM_2.5_ pollution concentrations of 0.0155 mg/m^3^ in the Boston area (Gold et al. 2000), where this study population is based. Although particulate air pollution also contains metals, the overall composition differs from welding fume. In addition, welding fume exposures occur intermittently over the course of hours, whereas air pollution exposures follow a different exposure pattern. Future research should investigate whether nighttime is also a susceptible period for particulate air pollution exposures. Likewise, the small sample size of the present study also limits study generalizability. Although we had sufficient power to link decreased night rMSSD and workday particulate exposures, we did not observe statistical significance among the other night measures. Also, we could not investigate effect modification. However, by restricting our sample, we were able to examine the sensitivity of our findings and found that the negative exposure–response relationship persisted after excluding workers who did not report either cardiac compromises or hypertension, suggesting that similar declines in nighttime HRV occurred in apparently asymptomatic healthy participants.

Our study is strengthened by internal validity and the cross-over study design. Each participant served as his own control and provided a baseline nonwork HRV measure over the same time period as work measures to account for individual cardiac and other risk factors. Potential confounders are limited to time-varying factors that may differ between work and nonwork periods, such as caffeine or alcohol consumption or noise levels. For the comparison of dichotomous exposure (work and nonwork), we were unable to account for these factors. However, for the exposure–response analysis, we hypothesize that neither caffeine and alcohol consumption or noise are correlated with daytime exposure; therefore, the potential for confounding bias is small. We adjusted for smoking by using a binary variable but were unable to account for the quantity of cigarettes consumed. However, workers were allowed to smoke on the job, and it is unlikely that the quantity of cigarettes consumed differed between work and nonwork periods. Thus, the likelihood of such a bias is low. Our study is strengthened by the detailed exposure assessment using real-time, personal PM_2.5_ exposure monitoring. We attempted to account for respirator use by adding an interaction term to the models of HRV at night (data not shown). Workers reporting no respirator use had a larger decline in HRV with workday PM_2.5_ exposure; however, the difference was not statistically significant. A limitation of the study is that potential co-pollutant gases including ozone, nitrogen oxides, or carbon monoxide were not measured. However, a previous investigation among this cohort of boilermakers performing welding during the overhaul of an oil-fired boiler, found low mean (± SD) time-weighted average concentrations of both ozone, 2.6 ±1.9 ppb and nitrogen dioxide 46.2 ± 1.7 ppb, and no correlations between PM_10_ and ozone or nitrogen dioxide (Liu et al. 2005). In addition, a previous investigation of shielded metal arc welding found minimal concentrations of carbon monoxide over a wide range of operating conditions (Burgess 1995). Likewise, the protective effect of respirator use suggests that particles (that can be captured by a respirator) rather than co-pollutant gases (that can pass through a respirator) are responsible for the observed changes in HRV.

## Conclusions

This panel study demonstrated decreases in long-duration HRV subsequent to metal-rich workday particulate exposure in a relatively healthy cohort of boilermaker construction workers. Furthermore, we found that the declines in HRV were most pronounced at night; this is consistent with results seen in studies of air pollution exposures (Pope et al. 1999). More research is needed to elucidate whether this is because nighttime incorporates the appropriate lag, captures the relevant sleep period, or is a period with less confounding. Finally, this study further supports the cardiotoxicity of metal-rich particulate exposure. Additional research is needed to elucidate which of the particulate metal constituents is responsible for decreases in nighttime HRV.

## Correction

The authors note that Yeatts et al. have recently published an article on the association between particulate exposures and long-duration HRV [Yeatts K, Svendsen E, Creason J, Alexis N, Herbst M, Scott J, et al. Coarse particulate matter (PM_2.5–10_) affects heart rate variability, blood lipids, and circulating eosinophils in adults with asthma. Environ Health Perspect 115:709–714 (2007)].

## Figures and Tables

**Table 1:** Population and exposure characteristics for male boilermaker construction workers (*n* = 36).

Characteristic	Mean ± SD or no. (%)
Age (years)^a^	41 ± 11
Range	22–63
Race	
White	29 (81)
Black	3 (8)
Hispanic	3 (8)
Asian	1 (3)
Current smoker^a^	12 (33)
Medical information^b^	
Hypertensive	6 (17)
Cardiac compromised	5 (14)
PM2.5 (mg/m^3^)^c^	0.73 ± 0.50
Range	0.04–2.70
Reported respirator use^c^	13 (26)

a At study entry.

b Ever reported condition at one or more study occasions.

c For 49 measurement occasions.

**Table 2:** Summary and comparison of long-duration HRV by nonwork and work periods.

	Nonwork^a^		Work^a^		
HRV measure (msec)	Mean ± SD	Range	Mean ± SD	Range	*p*-Value^b^
24-hr					
rMSSD	29.6 ± 13.9	7.6–71.1	26.9 ± 11.9	8.5–63.5	< 0.05
SDNN	130.9 ± 36.1	32.9–213.9	132.9 ± 37.5	39.0–224.7	0.65
SDNN_i_	56.3 ± 19.1	10.5–94.8	51.1 ± 16.7	11.9–90.5	< 0.01
Day^c^					
rMSSD	25.3 ± 10.8	8.3–60.7	22.0 ± 9.0	8.1–57.4	< 0.01
SDNN	98.8 ± 30.1	36.3–180.1	95.1 ± 27.1	36.8–190.5	0.37
SDNN_i_	52.4 ± 18.1	11.6–92.1	46.0 ± 15.1	13.9–89.0	< 0.01
Night^d^					
rMSSD	37.7 ± 19.7	6.5–98.2	34.4 ± 19.0	7.2–76.5	0.14
SDNN	101.3 ± 31.9	26.4–172.2	101.3 ± 33.5	28.8–202.0	0.99
SDNN_i_	65.0 ± 23.5	9.1–114.1	59.8 ± 22.5	8.2–123.6	< 0.05

a Thirty-six individuals and 49 measurements.

b Statistical comparison between work and nonwork measures using mixed regression models to account for multiple participation times.

c 0730–2130 hours.

d 0000–0700 hours.

**Table 3:** Effect estimates for change in long-duration HRV measures per 1-mg/m^3^ increase in workday PM2.5 exposures.

HRV measure	β^a^ (95% CI) (msec per 1 mg/m^3^)
24-hr	
rMSSD	−3.29 (−7.66 to 1.09)
SDNN	−11.66 (−28.84 to 5.51)
SDNN_i_	−4.58 (−10.16 to 0.99)*
Day	
rMSSD	−0.58 (−4.18 to 3.01)
SDNN	−9.39 (−22.34 to 3.57)
SDNN_i_	−4.06 (−9.22 to 1.10)
Night	
rMSSD	−8.32 (−16.29 to −0.35)**
SDNN	−14.77 (−31.52 to 1.97)*
SDNN_i_	−8.37 (−17.93 to 1.20)*

a Adjusted for nonwork HRV measure over the same time period, age, and smoking status.

* *p* < 0.10.

** *p* < 0.05.

**Table 4:** PM2.5 regression coefficients [95% confidence interval (CI)] for models of nighttime rMSSD, SDNN, or SDNN_i_ after restricting the data set.

HRV measure (msec)	Model I^a^,^b^ None	Model II^a^,^c^ No cardiac compromise	Model III^a^,^d^ No hypertension
Night rMSSD (msec)	−8.32 (−16.29 to −0.35)**	−8.19 (−17.31 to 0.93)*	−6.71 (−16.17 to 2.75)
Night SDNN (msec)	−14.77 (−31.52 to 1.97)*	−13.97 (−32.55 to 4.61)	−18.73 (−39.68 to 2.22)*
Night SDNN*_i_* (msec)	−8.37 (−17.93 to 1.20)*	−8.78 (−19.76 to 2.21)	−9.29 (−20.84 to 2.26)*

a Adjusted for nonwork nighttime HRV, age, and smoking status.

b Includes 36 individuals over 49 measurement occasions.

c Includes 33 individuals over 44 measurement occasions, including 2 individuals who reported no cardiac compromise on initial participation, and excluding subsequent participation reporting cardiac compromise.

d Includes 32 individuals over 41 measurement occasions, including 2 individuals who reported no hypertension on initial participation, and excluding subsequent participation reporting hypertension.

* *p* < 0.10.

** *p* < 0.05.
